# Electrophysiological differences between upper and lower limb movements in the human subthalamic nucleus

**DOI:** 10.1016/j.clinph.2019.02.011

**Published:** 2019-05

**Authors:** Gerd Tinkhauser, Syed Ahmar Shah, Petra Fischer, Katrin Peterman, Ines Debove, Khoa Nygyuen, Andreas Nowacki, Flavie Torrecillos, Saed Khawaldeh, Huiling Tan, Alek Pogosyan, Michael Schuepbach, Claudio Pollo, Peter Brown

**Affiliations:** a Department of Neurology, Bern University Hospital and University of Bern, Bern, Switzerland; b MRC Brain Network Dynamics Unit at the University of Oxford, Oxford, United Kingdom; c Nuffield Department of Clinical Neurosciences, University of Oxford, Oxford, United Kingdom; d Department of Neurosurgery, Bern University Hospital and University of Bern, Bern, Switzerland; e Department of Neurosurgery, John Radcliffe Hospital, Oxford University Hospitals NHS Foundation Trust, Oxford, United Kingdom

**Keywords:** Basal ganglia, Somatotopy, Motor network, Local field potentials, Directional deep brain stimulation

## Abstract

**Objective:**

Functional processes in the brain are segregated in both the spatial and spectral domain. Motivated by findings reported at the cortical level in healthy participants we test the hypothesis in the basal ganglia of Parkinson’s disease patients that lower frequency beta band activity relates to motor circuits associated with the upper limb and higher beta frequencies with lower limb movements.

**Methods:**

We recorded local field potentials (LFPs) from the subthalamic nucleus using segmented “directional” DBS leads, during which patients performed repetitive upper and lower limb movements. Movement-related spectral changes in the beta and gamma frequency-ranges and their spatial distributions were compared between limbs.

**Results:**

We found that the beta desynchronization during leg movements is characterised by a strikingly greater involvement of higher beta frequencies (24–31 Hz), regardless of whether this was contralateral or ipsilateral to the limb moved. The spatial distribution of limb-specific movement-related changes was evident at higher gamma frequencies.

**Conclusion:**

Limb processing in the basal ganglia is differentially organised in the spectral and spatial domain and can be captured by directional DBS leads.

**Significance:**

These findings may help to refine the use of the subthalamic LFPs as a control signal for adaptive DBS and neuroprosthetic devices.

## Highlights

• Beta desynchronization during leg movements involves higher beta frequencies.
• Limb specific spectral changes evident for contralateral and ipsilateral movements.
• Spatial distinction of limb-specific movements is evident at gamma frequencies.

## 1 Introduction

There has been considerable interest in the pathophysiological insights provided by recordings of local field potential (LFP) activity from electrodes implanted in the subthalamic nucleus (STN) during surgery for deep brain stimulation (DBS) in patients with Parkinson’s disease (PD). Studies have shown that beta (13–35 Hz) activity is exaggerated in patients who have been withdrawn from their antiparkinsonian medication, and this activity is suppressed by medication in proportion to the attendant clinical improvement (Kühn et al., 2006, Chen et al., 2010, Ozkurt et al., 2011). Likewise, DBS itself suppresses beta activity, and the degree of suppression correlates with the clinical improvement (Eusebio, 2011, Whitmer, 2012, Oswal, 2016, Trager, 2016). Some distinct functional roles have been attributed to the lower and upper beta frequency range, (Priori et al., 2004). Lower frequency beta activity has been reported to be most modulated by levodopa and to correlate most strongly with bradykinesia and rigidity (Priori et al., 2004, van Wijk, 2016), while higher beta frequencies are most coherent with cortical activity (Williams, 2002, Fogelson, 2006). However, to date, the functional relationships of the two oscillations remain unclear. Furthermore, basal ganglia beta activity is suppressed during voluntary movement, with some evidence that greater suppression is associated with improved motor performance (Doyle et al., 2005, Devos et al., 2006). STN gamma activity (40–100 Hz) increases with movement, as well as with dopaminergic therapy and is hence viewed as pro-kinetic signal (Cassidy et al., 2002, Brown, 2003, Androulidakis et al., 2006, Lalo et al., 2008).

It has been reported that, at the level of the cerebral cortex movement-related activity can be functionally segregated (Salmelin et al., 1995). Voluntary lower limb movements are associated with modulation at higher beta frequencies, whilst upper limb movements are associated with modulation of lower beta frequencies (Neuper and Pfurtscheller, 2001, Praamstra, 2006, Heideman et al., 2015). Similarly, an increased activity in the higher beta frequency range has been described over leg motor and nearby supplementary motor cortical areas in a recently published atlas of normal intracranial EEGs (Frauscher et al., 2018). These observations suggest the hypothesis that the two beta activities recorded in the STN similarly relate to upper and lower limb motor circuits. Here we explicitly test this hypothesis by recording LFP activity from the STN while PD patients make voluntary movements of the upper or lower limb.

## 2 Methods

### 2.1 Patients and surgery

We investigated the movement-related modulation of STN LFPs during upper and lower limb movement in 12 consecutive PD patients undergoing STN DBS surgery to improve motor symptoms (see Supplementary Table 1). Recordings were performed as part of our routine intra-operative electrophysiological assessment from both hemispheres, except in 4 subjects (cases 5, 6, 7 and 10) in whom LFPs were recorded in one hemisphere only, leading to a total of 20 hemispheres. The patients were off their normal dopaminergic medication during the recording. All patients were operated at the University Hospital Bern and the local ethics committee approved the study (2017-00551). Patients were implanted with directional leads and the Vercise PC (Boston Scientific). The contacts of the directional DBS leads are distributed on 4 levels along the vertical axis. Level 2 and 3 contain three segmented contacts (non-circular), allowing stimulation focussed in 3 different directions (at 120° angles), while levels 1 and 4 consist of a single ring/omnidirectional contact. The DBS target was localised using the T2-sequence of the pre-operative 3T MRI and preoperative stereotactic CT-scan (with Leksell G frame) using Brainlab Elements software (Brainlab AG, Germany). Intraoperative targeting was optimised by microelectrode recordings and selective test stimulation.

### 2.2 Postoperative localisation of directional contacts

The Lead-DBS Matlab toolbox version 2.0.0.6 was used for DBS lead visualisation (Horn and Kühn, 2015). To this end preoperative MRI and postoperative CT scans were co-registered using SPM12 (Statistical Parametric Mapping 12; Wellcome Trust Centre for Neuroimaging, UCL, London, UK) and normalised into the MNI 152 2009b space (Montreal Neurological Institute) (Avants et al., 2008). Using the *Precise and Convenient Electrode Reconstruction for Deep Brain Stimulation* (PaCER) toolbox, the DBS lead was pre-localised and then manually adjusted if necessary (Husch et al., 2018). The final three-dimensional coordinates of each directional contact were then projected into the DISTAL Atlas, a subcortical atlas based on multimodal MRI, histology and structural connectivity that displays basal ganglia structures, for further somatotopic analyses (Ewert et al., 2018) for further somatotopic analyses. All directional contacts from both hemispheres were projected onto the right DBS template by using a non-linear flip function (Lead-DBS Matlab toolbox).

### 2.3 Local field potential recordings and limb assessment

**Fig. 1:**
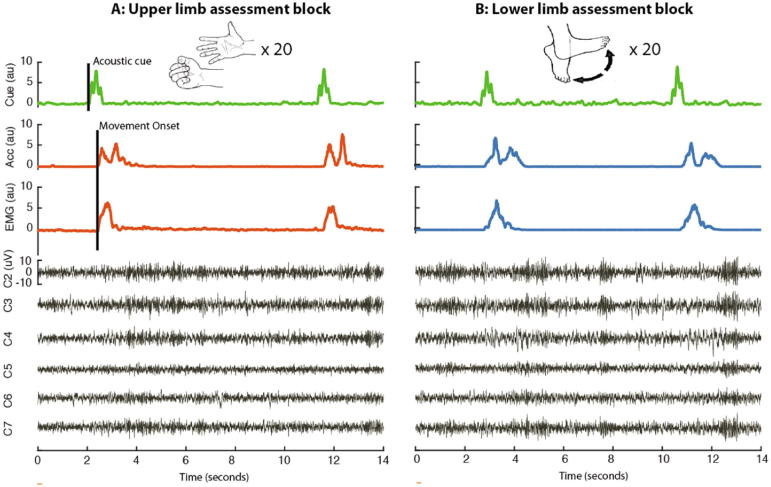
Intraoperative assessment. Assessment of repetitive, cued upper (A) and lower (B) limb movements during simultaneous LFP recording. Surface EMG electrodes were placed on the upper limb (forearm flexor muscles) and lower limb (tibialis anterior). Accelerometers were additionally placed on the hand and foot to better delineate task-related movements. Patients were then asked to perform a block each of contralateral upper and lower limb movements followed by blocks of ipsilateral upper and lower limb movements. The upper limb movement consisted of closing and opening of the hand, while the lower limb movement involved foot dorsi-extension and then plantar flexion. Each movement was preceded by a verbal go cue and the mean inter-trial interval was 7.9 s ± 0.15. On average 16.1 ± 0.37 movements were collected within each block. LFPs were recorded simultaneously from the six directional contacts.

LFPs were recorded during DBS surgery from the six directional contacts (contacts 2–7, Fig. 1) after the lead was placed in its final position. Recordings were performed with a TMSi-Porti amplifier (Twente Medical Systems International, Netherlands) using a sampling frequency of 2048 Hz and common average referencing. Surface EMG electrodes were placed on the upper limb (forearm flexor muscles) and lower limb (tibialis anterior). Accelerometers were additionally placed on the hand and foot to improve detectability of the onset of task-related movements. After a brief recording at rest (mean duration: 99.2 s ± 8.5 s), patients were asked to perform a block each of contralateral upper and lower limb movements followed by ipsilateral upper and lower limb movements. They were informed of the desired response at the beginning of each block, and instructed that only this movement should be made in response to auditory go cues. The upper limb movement consisted of closing and opening of the hand, while the lower limb movement involved foot dorsi-extension and then plantar flexion (Fig. 1). Note that whether the assessment began with upper or lower limb blocks was randomised to avoid any order effect. Each single movement was prompted by a verbal ‘go’ command recorded with a microphone and the inter-trial time was around 7.9 s ± 0.15 s [range: 6.0–11.3 s]. This ensured that beta activity, which rebounds after movement completion (Pfurtscheller and Lopes da Silva, 1999), did not compromise the baseline period of the next movement. We aimed to record 20 trials in each of the 4 blocks. The precise number of blocks and trial numbers was allowed to vary because of the intraoperative setting and associated constraints. Ipsilateral upper and lower limb movement blocks were recorded in only 13 hemispheres because of intraoperative time constraints (see Supplementary Table 1).

### 2.4 Signal processing

The raw signal was down-sampled to 300 Hz and high-pass filtered at 1 Hz. Frequency decomposition was performed at 1 Hz resolution using the Wavelet method (ft_specest_wavelet script in Fieldtrip – Morlet Wavelet, width = 10, gwidth = 5; Donders Institute for Brain, Cognition and Behaviour, 2010). For each hemisphere the directional contact with the highest normalised (relative to its sum) resting beta amplitude (13–35 Hz) was selected to determine the average resting amplitude-frequency spectrum, which is illustrated in Supplementary Fig. 1. For two hemispheres (1, 13), 1 directional contact had to be excluded from the analysis due to saturation during the recording.

For the movement related signals, the continuous signal was segmented into 4 s blocks either centred around the movement onset or end of the movement, which were defined visually according to the rectified, detrended and smoothed (0.1 s) EMG and accelerometer signals. Each single trial was visually inspected and those containing artefacts were removed, leaving an average number of 16.1 ± 0.37 [range: 9–25] trials per block for further analyses. The average delay between the onset of this auditory cue and movement onset was 0.43 s ± 0.02 [range: 0.17–1.35 s].

Event-related power changes were estimated by normalizing the data with respect to the baseline power averaged in a pre-cue window ranging from −2s to −1.5 s before movement onset (and hence, given the reaction times, also preceding the imperative cue) for each corresponding movement block. With regard to movement-related modulation, we considered the beta power (13–35 Hz) event-related desynchronization (ERD) and post-movement event-related synchronization (ERS) as well as the gamma (40–85 Hz) ERS. The beta ERD and gamma ERS were quantified as the percentage spectral change in a common, representative time-window, i.e. start of the movement until 300 ms after movement onset, while the beta ERS was quantified from the end of the movement to 1 s after the end of the movement. To control whether results depended on this arbitrary selection of the time-windows, a further analysis was performed by applying a 300 ms time-window, around the time point of the average maximum beta ERD/ERS and gamma ERS, separately for upper and lower limb movements. Note, for these comparisons we considered the same directional contact pair for both upper and lower limb modulation. To this end we first averaged the ERDs for upper and lower limbs and secondly selected the directional contact pair with the greatest common ERD. This step was necessary to avoid a systematic bias in favour of upper or lower limb modulation through channel selection.

### 2.5 Spatial-electrophysiological processing

Each directional contact was characterised by its xyz-coordinates and the degree of modulation in a given frequency bin during upper and lower limb movements. First, independently of the underlying anatomy, we investigated whether the directional contacts that showed the highest relative degree of modulation (ERD for frequencies <45 Hz and ERS for frequencies >45 Hz) for upper and lower limb movements matched or whether they were distinct. Second, we projected the directional contacts with the greatest movement-related modulation on the anatomical STN from the Distal atlas (Ewert et al., 2018) and tested whether the neuronal activation of upper and lower limb movements could be spatially discriminated in this common space. To this end, we adopted a multivariate kernel density estimation technique (Silverman, 2018) to estimate the probability density function (pdf) of the 20 segmented contacts that showed the maximum modulation separately for both the upper and lower limb. The 20 coordinates were weighted by the degree of movement-related modulation to give less weight to those coordinates with smaller modulation, which we assumed reflected the source of movement-related activity less accurately. Such an approach allowed us to estimate and visualize the distribution of contacts in this common space. To quantify any difference between the pdfs for the upper and lower limb, we used the Monte Carlo method drawing a large number of samples (N = 10,000) from these distributions in order to obtain a reliable estimate of the mean coordinate for both the upper and lower limb. This procedure was repeated for each frequency bin from 13 to 85 Hz. As a result we obtained three data-series (one each for the x, y and z coordinates) across frequencies with the expected localisation of the neuronal modulation for both upper and lower limb movement in each plane. These were used for further analyses.

### 2.6 Statistical analyses

Statistical analyses were performed using Matlab (version R 2015b; MathWorks, Natick, MA). All data are presented as means ± standard error of the mean (SEM). To evaluate the statistical difference between movement-related spectral changes and differences in expected pdfs for upper and lower limb movement we used a cluster-based permutation procedure to correct for multiple comparisons. P-values were derived by randomly permuting the assignment of condition labels for all hemispheres 2000 times. For each frequency point the z-statistic of the actual mean difference was computed based on the distribution of the 2000 differences resulting from permutation. The resulting P-values were then corrected for multiple comparisons as follows: Suprathreshold clusters (pre-cluster threshold: P < 0.05) were determined for each permutation, and the sum of the absolute z-statistics within these clusters was stored to form a distribution of the largest suprathreshold-cluster values. Finally, the 95th percentile of this distribution served as statistical threshold for the map of the actual absolute z-statistics of the real difference (Maris and Oostenveld, 2007). Thus only those significant clusters that exceeded the threshold survived the multiple comparison correction. To test whether the degree of modulation of upper and lower limb movement was similarly distributed across the directional contacts within the DBS lead, we performed a Spearman correlation of their movement-related modulation for each frequency bin (13–85 Hz). R-values were then Fisher-Z transformed and averaged.

## 3 Results

### 3.1 Beta ERD (increase) related to contralateral upper and lower limb movements

**Fig. 2:**
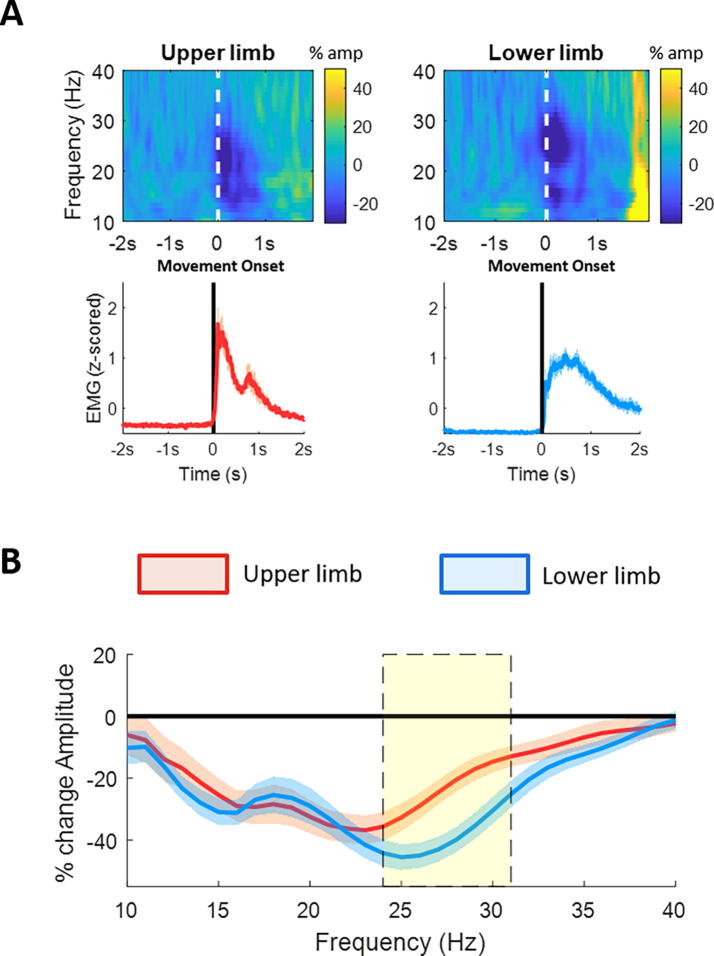
Beta ERD related to contralateral upper and lower limb movements. (A) upper panel shows the averaged time frequency spectra of the single common directional contact with greatest modulation (ERD) in the beta frequency band (13:35 Hz). All single trials were aligned to the movement onset (vertical line). The lower panel shows the corresponding normalised and averaged EMG for upper limb (forearm flexor muscles) and lower limb (tibialis anterior) movements. (B) shows the percentage movement-related spectral amplitude change in the beta frequency band for upper (red) and lower limb (blue). This was calculated as percentage change between the movement period (i.e. movement onset to 0.3 s after movement onset) and the baseline period (−2 s to −1.5 s) before movement onset. Lower limb movements involve a significantly stronger ERD in the higher beta band, in particular in the frequency range between 24 and 31 Hz (yellow shaded area). Values are mean ± SEM. (For interpretation of the references to color in this figure legend, the reader is referred to the web version of this article.)

**Fig. 3:**
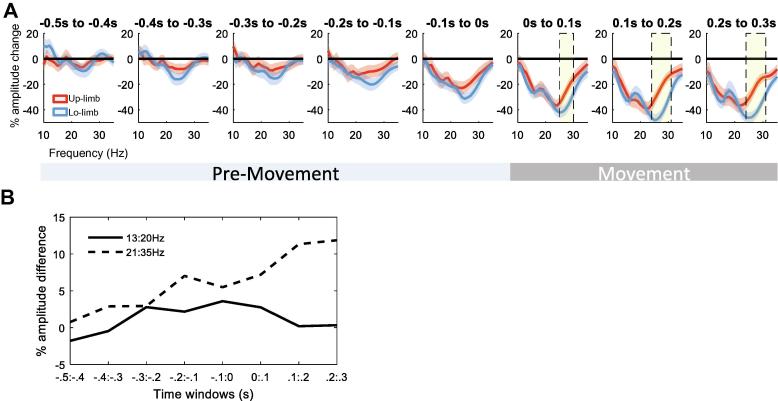
Evolution of beta ERD for upper and lower limb movements: (A) shows the evolution of the beta modulation in time windows of 100 ms beginning from 500 ms before movement onset to 300 ms after movement onset. The beginning of the beta ERD difference can already be seen in the windows before movement onset. The beta ERD around 0.2 s before movement onset shows a trend for stronger deflection in the higher beta band for the lower as opposed to the upper limb movement. However, according to cluster-based permutation test the difference becomes significant only with onset of the movement (yellow shaded areas). Values are illustrated as mean ± SEM. (B) shows the evolution of the averaged amplitude differences between the upper and lower limbs in the lower (black solid line) and higher beta frequency range (black dotted line). It illustrates the growing distinction in the higher beta band, already evident in the pre-movement period, whereas the amplitude difference in the lower beta band between the limbs is marginal and fluctuates around zero.

Fig. 2 shows the spectral changes in the beta frequency range for upper and lower limb movements relative to the baseline (see Methods). Here, for each individual hemisphere the directional contact with the greatest modulation in the beta frequency band (13–35 Hz) common to both limbs was selected. The cluster-based permutation test shows that beta power in the higher frequency range (24–31 Hz) was more strongly suppressed during lower limb compared to upper limb movements. Note, the stronger suppression of higher frequency beta activity during lower limb movements was unlikely related to greater muscle activity during foot movements, as there was no correlation between 24 and 31 Hz beta ERD and the mean rectified EMG of the same time window during contralateral foot movements, both within and across hemispheres (second-level analysis: mean ± SEM Spearman’s rho = 0.06 ± 0.08, p = 0.46 (*one sample t-test*); correlation across recordings: Spearman’s rho = −0.014, p = 0.96, respectively). The results were similar with the spectral change derived from alternative time windows, separately for upper and lower limb movements (Supplementary Fig. 2). In a further related analysis we determined whether the difference in the mean beta ERD over 24–31 Hz between upper and lower limb movements already arose before movement onset. We considered a difference that was already present before movement initiation would be unlikely to be due to a difference in the vigour with which the movement was executed. Accordingly, the change in mean beta ERD over 24–31 Hz was plotted for upper and lower limb movements in 100 ms time windows starting from 500 ms before movement onset to 300 ms after movement onset. This showed that the difference in the beta ERD over 24–31 Hz between upper and lower limb movement built-up before movement onset, whereupon it became significant (Fig. 3).

### 3.2 Beta and gamma ERS (decrease) related to contralateral upper and lower limb movements

**Fig. 4:**
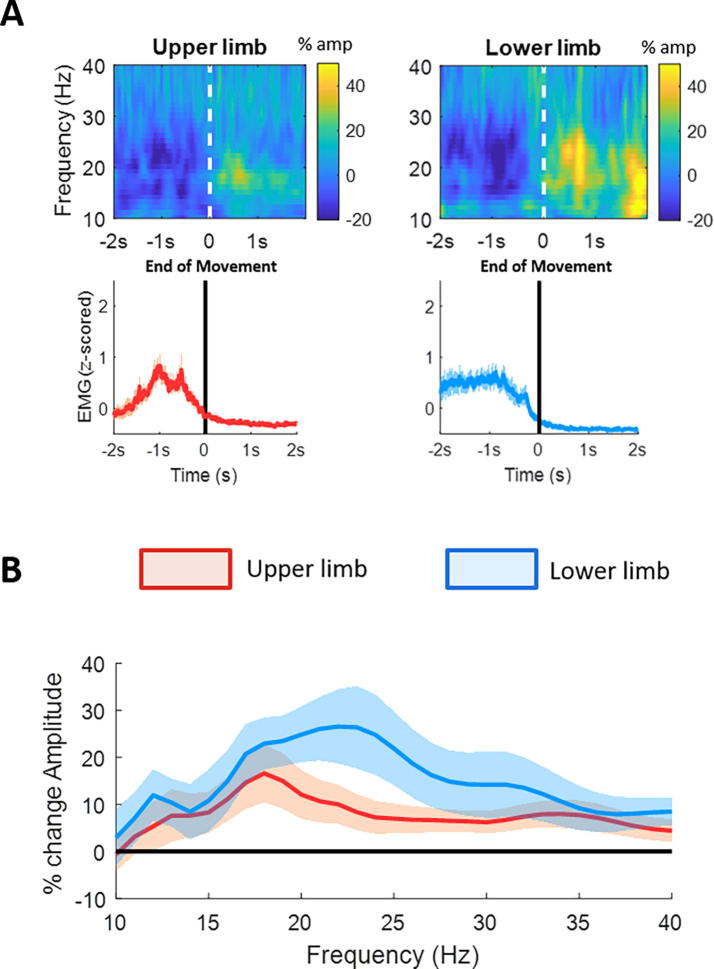
Beta ERS related to contralateral upper and lower limb movements. (A) Upper panel shows the averaged time frequency spectra of the single common directional contact with greatest modulation (ERD) in the beta frequency band (13:35 Hz). All single trials were aligned to the end of the movement (vertical line). Lower panel shows the corresponding normalised and averaged EMG for upper limb (forearm flexor muscles) and lower limb (tibialis anterior) movements. (B) shows the percentage movement-related spectral amplitude change in the beta frequency band for upper (red) and lower limb (blue). This was calculated as percentage change between the movement period (i.e. end of movement to 1 s after the end of movement) and the baseline period (−2 s to −1.5 s before movement onset). There is a trend for beta ERS at higher frequencies for lower limb movements, however no significant difference on cluster-based permutation test was found. Lines depict means ± SEM. (For interpretation of the references to color in this figure legend, the reader is referred to the web version of this article.)

**Fig. 5:**
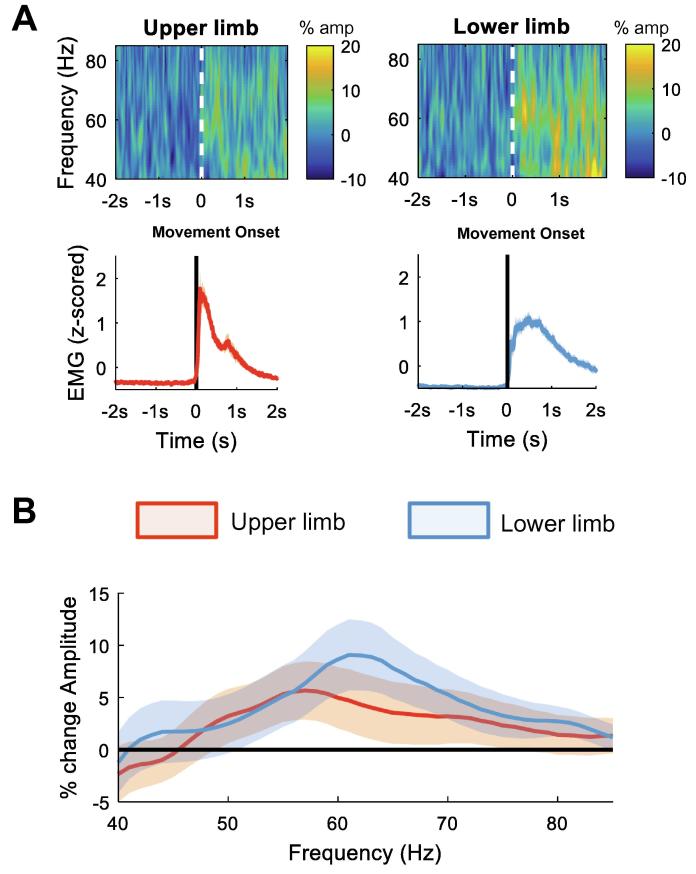
Gamma ERS related to contralateral upper and lower limb movements. (A) The upper panel shows the averaged time frequency spectra for the gamma frequency range (40:85 Hz) of the common single directional contact with greatest modulation (ERD) in the beta frequency band (13:35 Hz) (same contact as in previous figures). All single trials were aligned to the movement onset (vertical line). The lower panel shows the corresponding mean normalised EMG for upper limb (forearm flexor muscles) and lower limb (tibialis anterior) movement. (B) shows the percentage movement-related spectral amplitude change in the gamma frequency band for upper limb (red) and lower limb (blue). This was calculated as percentage change between the movement period (from 0 to 0.3 s after movement onset) and the baseline period −2 s to −1.5 s before movement onset. No significant difference in the gamma ERS was found. Lines depict means ± SEM. (For interpretation of the references to color in this figure legend, the reader is referred to the web version of this article.)

Fig. 4 illustrates the change in beta activity for the same trials as above but now aligned to the end of the movement. In line with the spectral characteristics of the ERD for contralateral movements, the post-movement beta ERS also showed a greater modulation for the lower limb movements at higher beta frequencies compared to the upper limb movements. However, the difference was no longer significant when correcting for multiple comparisons with the cluster-based permutation test. Fig. 5 shows the movement-related gamma ERS for upper and lower limb movements for trials aligned to the onset of the movement. No significant difference was found. Moreover, ERS results were not different when a separately optimised time window for upper and lower limb movements was selected (Supplementary Fig. 2).

### 3.3 LFP spectral modulation related to ipsilateral upper and lower limb movements

**Fig. 6:**
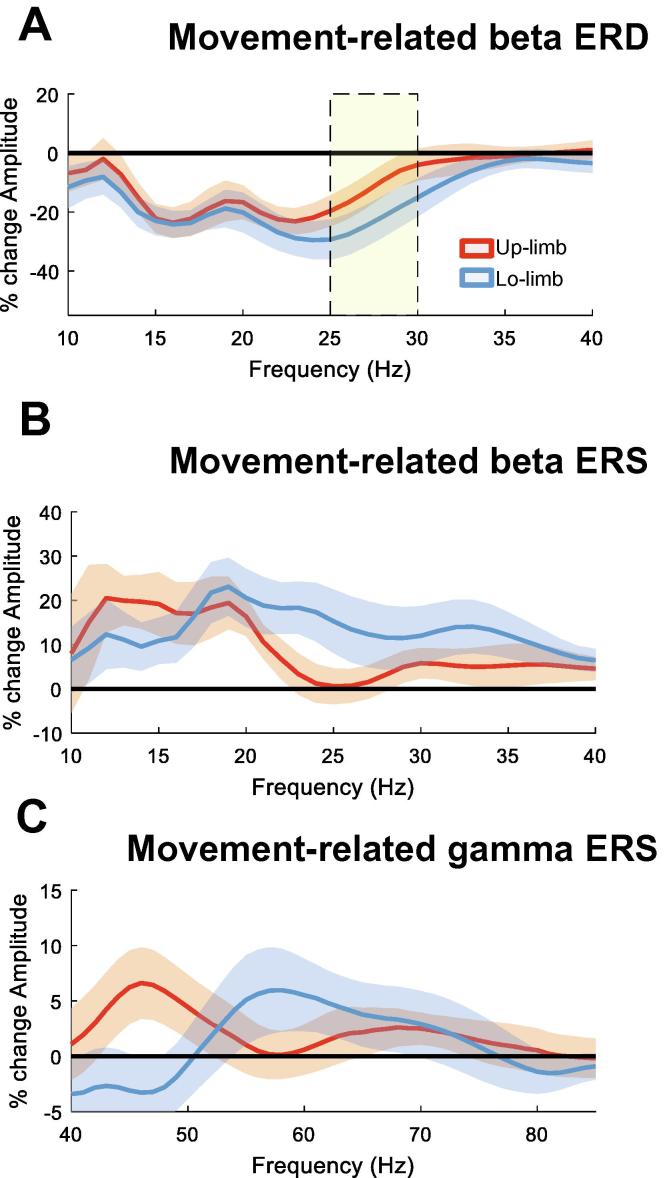
Beta ERD/ERS and gamma ERS related to ipsilateral upper and lower limb movements. (A) shows the movement-related beta ERD for the ipsilateral upper and lower limb. As previously shown for the contralateral limb movements, the ERD for lower limb movement is significantly greater at higher beta frequencies (24-29 Hz, yellow shaded area) than with upper limb movement. (B) shows the movement-related beta ERS for the ipsilateral upper and lower limbs. No significant difference was identified by the cluster-based permutation test, nevertheless, there was a trend for greater rebound at higher beta frequencies for the lower limb movements (similar region as shaded area in the upper panel). (C) shows the movement-related gamma ERS for the ipsilateral upper and lower limbs. No significant difference was found in the cluster-based permutation test. Lines depict means ± SEM. (For interpretation of the references to color in this figure legend, the reader is referred to the web version of this article.)

In 13/20 hemispheres, the movements of upper and lower limbs ipsilateral to the STN were recorded and key analyses repeated. Fig. 6A illustrates the beta ERD for ipsilateral upper and lower limb movements. This again revealed a significantly greater involvement of higher beta frequencies (24–29 Hz) for lower limb movements as opposed to upper limb movements. Additionally, although not significant, in higher beta frequencies the beta ERS again was stronger for lower limb movements compared to upper limb movements (Fig. 6B). There was no difference with respect to the gamma ERS (Fig. 6C). As above, results were maintained with alternative time-window selection (Supplementary Fig. 2).

### 3.4 Spatial distribution of spectral changes related to upper and lower limb movements across DBS electrodes

**Fig. 7:**
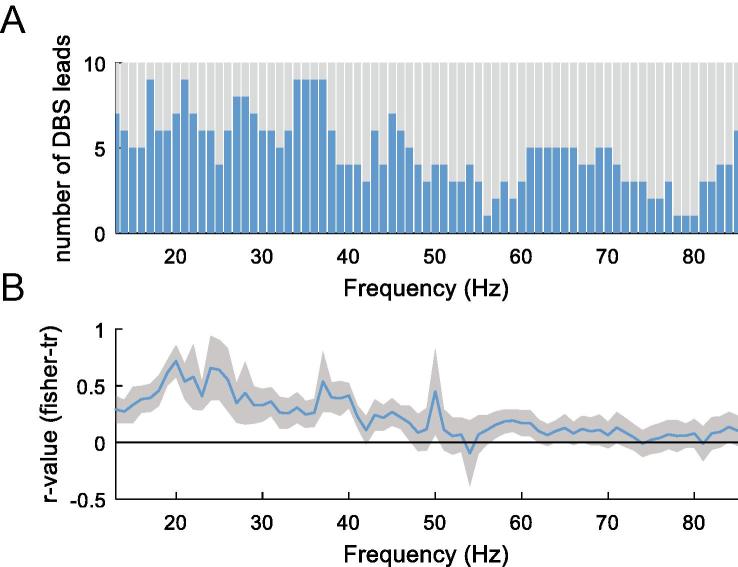
Spatial distribution of spectral changes related to upper and lower limb movements across electrodes*.* (A) illustrates the number of DBS leads (maximum n = 20) in which the maximal modulation at each frequency (13–85 Hz) occurred at the same contact for upper and lower limb movements. Contacts were more likely identical for beta frequencies (13–35 Hz: 6.7 ± 0.3 identical contacts) than for gamma frequencies (55–85 Hz: 3.4 ± 0.3 identical contacts), p < 0.001 (*ranksum test*). (B) correlations between the degree of modulation of all directional contacts during upper vs. lower limb movements. Illustrated are the Fisher’s Z-transformed and averaged Spearman’s correlation coefficients across the same frequency range as above. Similar as in (A), a relatively high correlation, i.e. less spatial discrimination within the DBS lead, was found for modulation at beta frequencies, and more spatial heterogeneity for the modulation at gamma frequencies where r-values dropped to near zero. Lines depict means ± SEM.

Fig. 7A illustrates separately for each frequency the number of DBS leads in which the contacts that showed the maximal modulation for contralateral upper and lower limb movements were the same. The figure shows a greater overlap of maximally reactive contacts during upper and lower limb movements for beta (13–35 Hz: average matching in 6.7 ± 0.3 DBS leads) than for gamma frequencies (55–85 Hz: matching in 3.4 ± 0.3 DBS leads), p < 0.001 (*ranksum test*). In a further analysis we included all 6 directional contacts and correlated their degree of modulation during upper limb movements with that during lower limb movements (Fig. 7B). This indicated a similar pattern whereby the spatial distribution of movement-related LFP modulation across directional contacts was more similar for upper and lower limbs over the beta than gamma band.

### 3.5 Spatial distribution of spectral changes related to upper and lower limb movements within the STN

**Fig. 8:**
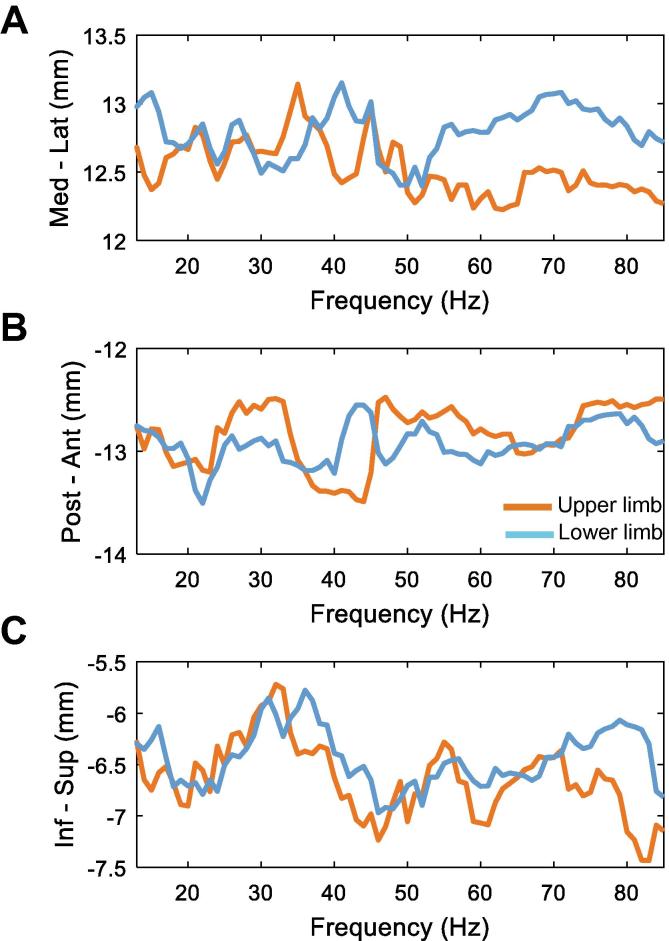
Spatial distribution of upper and lower limb movement-related modulation across axes within the STN. A/B/C show the expected value of the weighted probability density functions for upper (red) and lower limbs (blue) across the different frequencies for all the three axes. Similar as in Fig. 7, the spatial discrimination within the STN between upper and lower limb modulation is stronger for activity at higher frequencies. In particular, the contacts with the strongest lower limb movement-related gamma modulation were more lateral (A, 55–85 Hz) and superior (C, 70–85 Hz) than the contacts with the maximum upper limb-related modulation. (For interpretation of the references to color in this figure legend, the reader is referred to the web version of this article.)

The above results at the level of the DBS electrode raised the question whether the spatial distribution of neuronal activity differed between upper and lower limb movements in a frequency-specific way at the level of the STN. To test this, we separately calculated the probability density function (pdf) of the coordinates obtained from the contacts showing the highest modulation during upper and lower limb movements. The coordinates were weighted by the degree of relative modulation. Fig. 8 shows the expected values of the pdf for each frequency and axis. Spatial discrimination seems to be more pronounced in higher frequencies. The contacts that showed the strongest lower limb movement-related gamma modulation were more lateral (A, 55–85 Hz) and superior (C, 70–85 Hz) than the contacts with the maximum upper limb-related modulation.

**Fig. 9:**
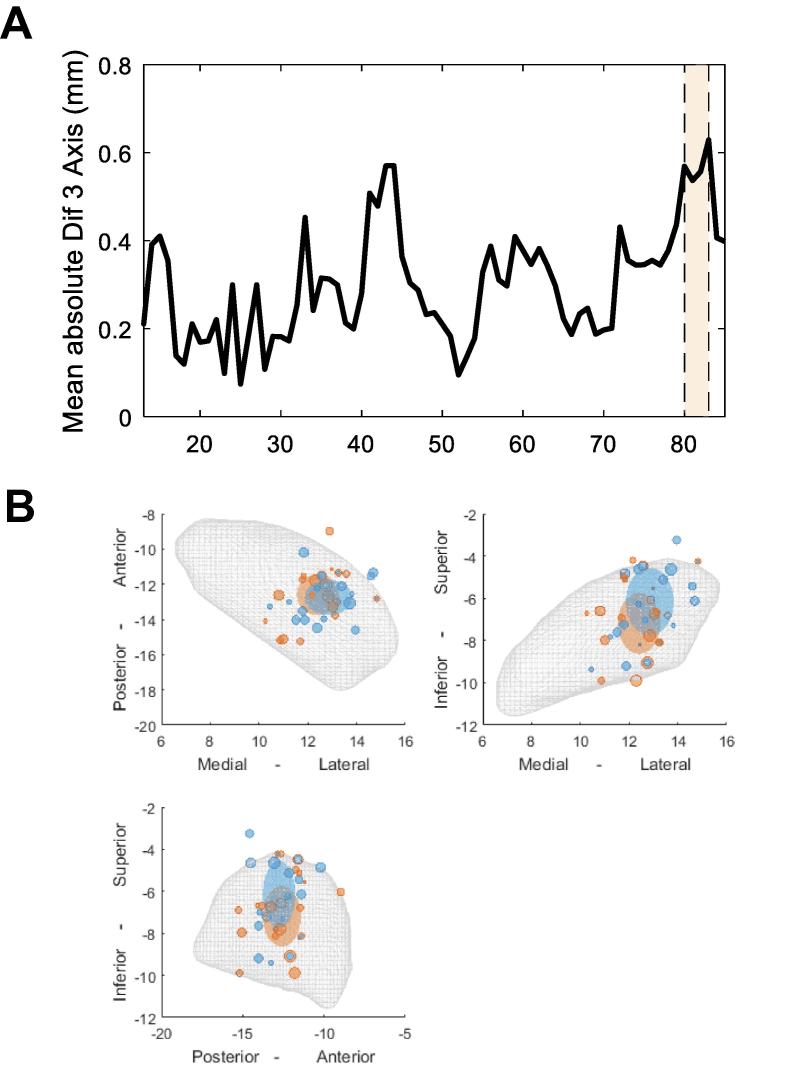
Spatial distribution of upper and lower limb activity within the STN. (A) shows the absolute difference of the expected value of the pdf for upper and lower limbs based on the contacts with the highest modulation and averaged across the 3 axes. The significant cluster between 80 and 83 Hz shows that this activity was relatively spatially separated when comparing upper vs. lower limb movement. (B) shows the example for the localisation of the directional contacts with the strongest 80 Hz modulation for upper (red) and lower (blue) limb movements relative to the STN (grey mesh) in three different planes. The large ellipsoids illustrate the expected values from the pdfs. Their diameter corresponds to the mean distance of the contacts. The biggest shift is that the blue ellipsoid, which represents the spatial centre of lower limb modulation, is more superior and lateral compared to the ellipsoid representing sites showing the maximal modulation during upper limb movements. (For interpretation of the references to color in this figure legend, the reader is referred to the web version of this article.)

Finally, to test which frequencies of modulation involve significant spatial selectivity we calculated the absolute difference of the expected values of the two pdfs for upper and lower limbs averaged across the 3 axes (Fig. 9A). We then applied a cluster-based permutation test, between the effective measured absolute difference and the absolute difference obtained by shuffling the upper and lower limb labels and repeating the algorithm (1000 iterations). This revealed a significant cluster from 80 to 83 Hz where upper and lower limb modulations were spatially discriminable. Fig. 9B shows the contacts with the best upper and lower limb modulation at 80 Hz projected on to the anatomical STN template. The centre of lower limb gamma modulation tends to be more superior and more lateral relative to the hotspot of upper limb modulation at this frequency.

## 4 Discussion

We set out to test the hypothesis that lower and higher beta band activity is modulated differently by voluntary upper and lower limb movements. Although movement of both limbs modulated beta activity in the lower beta band to a similar degree, lower limb activation was characterised by clearly greater modulation of higher beta frequencies. This observation was true for both contra- and ipsilateral lower limb movements.

The simplest explanation for the above findings is that they are a natural consequence of the preservation of somatotopic representations around cortical-basal ganglia loops (Alexander and Crutcher, 1990; Nambu, 2011), so that the somatotopy-related spectral differences seen at the cortical level are also reflected subcortically. Thus the physiological lower frequency beta activity modulation seen over more lateral motor cortex related to upper limb representations, and the higher frequency beta activity modulation seen over mesial motor cortex related to lower limb representations (Neuper and Pfurtscheller, 2001), is also retained at the level of the STN. The recent observation that stepping is accompanied by rhythmic amplitude modulation of beta activity in the high beta band would be in keeping with this suggestion (Fischer et al., 2018). Yet, the above interpretation of our findings is not entirely satisfying, as differences in the frequency of peak modulation of beta related to the limb activated are limited to the post-movement rebound at the cortical level, but in the STN are even more distinct during the beta suppression that precedes and accompanies movement (Neuper and Pfurtscheller, 2001). Rather our results suggest that lower limb movements involve the greater recruitment of additional networks resonant at higher frequencies, while core networks characterised by low frequency beta synchrony in the STN seem to be involved to a similar extent during both upper and lower limb movements (Salmelin et al., 1995, Wheaton et al., 2008). A clue to the nature of these additional networks is afforded by studies of STN-cortical coherence, which demonstrate that the higher beta band LFP activity in the STN is particularly coherent with cortical activity over a mesial region that includes the leg area of primary motor cortex, but also mesial premotor areas (Oswal et al., 2016). Subdural recordings in otherwise healthy epileptic patients confirm that both unilateral upper and lower limb movements are preceded and accompanied by activity in the bilateral supplementary motor cortex (Ikeda et al., 1992). Thus we propose the working hypothesis that upper limb movement in PD involves modulation of STN activity in the lower beta band, whereas that lower limb movement entails the additional modulation of STN activities related to associative motor loops which are characterised by resonances in the high beta band.

However, an alternative explanation should also be considered. Could the networks supporting movement of the upper and lower limb be similar in their organisation and spectral characteristics, with the difference identified here instead being due to a difference in the effort made in the two movements? Previous reports suggest that the level of beta desynchronization at the cortical level is relatively independent of the effort, load or speed of voluntary movements (Kilavik et al., 2013, Nakayashiki et al., 2014). Indeed, the cortical ERD can be recorded during motor-related activities that do not require a force output, such us action observation (Babiloni et al., 2002, Koelewijn et al., 2008), passive movement or motor imagery (McFarland et al., 2000, Nakagawa et al., 2011). There is some evidence that the desynchronization in the low beta band recorded in the subthalamic nucleus after upper limb movement onset may scale with low degrees of effort (Tan et al., 2015). However, if this were the explanation for the decreased high frequency beta during lower limb movements then we would have expected to also see a decrease in the low frequency beta during lower limb movements and this was not the case. Furthermore, in the current study we saw the differential reactivity in the high beta band build up prior to movement onset. In addition to mitigate against this confound, we explicitly sought a correlation between the 24 and 31 Hz beta ERD and the mean rectified EMG during the same time window when making contralateral foot movements, and found none, suggesting that this activity was not dependent on effort.

### 4.1 Spatial segregation of upper and lower limb activities in STN

Our findings reveal that lower limb movement-related modulation in the beta frequency range (13–35 Hz) involves more reactivity at higher frequencies (24–31 Hz) compared to upper limb movement-related beta modulation. In contrast, upper and lower limb movements were not associated with discernible spatial distributions within this frequency band. Previously, we have shown that directional electrodes have a high enough spatial resolution to detect differences in the distribution of beta activity recorded at rest that allow prediction of the optimal stimulation site (Tinkhauser et al., 2018). Thus, our combined results suggests that although beta activity seems to reflect the distance to the sensorimotor region (Zaidel et al., 2010, Horn et al., 2017), beta modulation is not spatially specific enough to distinguish between lower limb and upper limb regions. However, a spatial segregation was detectable for activity in the gamma band (80–83 Hz), which is generally in line with the observation that movement-related gamma modulation is more focal than slower rhythms (Donner et al., 2009, Wang, 2010). In our cohort of patients, gamma activity related to lower limb movements was localised slightly more lateral and superior compared to that for upper limb movements. This contrasts with the anatomical distribution derived from non-human primate and patient studies (Rodriguez-Oroz et al., 2001; Theodosopoulos et al., 2003; Nambu, 2011), although it must be noted that these latter studies explore the distribution of single neuronal discharges and not the synchronised activity of local gamma band networks. The latter are picked up with very different electrodes. In addition, local field potential activity is believed to reflect synchronised input to the nucleus, whilst single neuron discharges represent output activity (Buzsáki et al., 2012). Although this distinction may not be so important in rodents where interneurons may be absent in the subthalmic nucleus, evidence suggests the existence of an interneuronal population within the nucleus in the human (Levesque and Parent, 2005). We also cannot discount the possibility that our gamma band distributions were biased by stun effects (Chen et al., 2006). Furthermore, in both PD patients and MPTP (1-methyl-4-phenyl-1,2,3,6-tetrahydropyridine) models of PD in non-human primates, spatial selectivity in the STN is reduced in comparison to healthy-non human primates (Wichmann et al., 1994, Rodriguez-Oroz et al., 2001, Romanelli et al., 2005, Tankus et al., 2017). Spatial selectivity can be partially recovered by administration of apomorphine, suggesting that selectivity may be particularly impaired in patients recorded OFF medication, as was the case here (Levy et al., 2001). Considering that patients were recorded OFF medication, when finely-tuned gamma activity generally seems to be reduced (Brown, 2003, Brown et al., 2001, Litvak, 2011), the present data may under-estimate the degree of spatial segregation to be expected in the on-drug state.

### 4.2 Why might segregation of activities in the spatial and spectral domain be important?

We found evidence for the partial segregation of motor processing streams related to upper and lower limb movements in the spectral and spatial domains. Segregation and integration within and across networks are believed to be important organisational features facilitating the adaptive control of neural activity (Tononi et al., 1994, Mohr et al., 2016). The consequence of disturbed neuronal segregation can for instance be seen in dystonia which is clinically characterised by co-activation of muscle agonists and antagonists and overflow movements, as well as enlarged sensory receptive fields (Vitek et al., 1999, Chiken et al., 2008, Nambu, 2011). Information flow within the cortical-subcortical motor loop is not organised in entirely segregated channels, but rather involves parallel processing with some degree of convergence (Nambu, 2011). Nevertheless, in the healthy state, the topographic distinction between supplementary motor areas and primary motor areas and between body parts is well preserved in the cortical-subcortical motor loop. This has been hypothesised to allow differentiated motor control with high spatial precision (Nambu, 2011). Different spectral characteristics of sub-loop activities could further promote effective and selective neuronal communication (Singer, 2018).

Spectral and spatial distinctions related to upper and lower limb movements may also be of potential clinical relevance. They could, for instance, help to guide surgical implantation or programming of DBS leads where symptoms differentially affect upper or lower limbs. Attention to the different beta sub-bands might also potentially increase the information available for the control of adaptive DBS, or even of neuroprostheses (Tan et al., 2016, Shah et al., 2018).

These translational implications are speculative, however, and it is worth stressing the limitations of the present study. The acquisition of LFPs took place intraoperatively directly after microelectrode recording (1–3 trajectories) was performed and therefore a confounding stun effect cannot be excluded (Koop et al., 2006). Moreover, motor assessments were limited by intraoperative time constraints and patient fatigue.

## 5 Conclusion

Here we provide evidence that at the level of the motor network indexed by LFP changes the STN not only exhibits some topographic segregation between areas involved in the processing of upper and lower limb movements, but also involves partial segregation of these activities in the frequency domain. These differences can be captured and potentially exploited by segmented directional DBS leads.
