# Supplementary material for: Electrophysiological differences between upper and lower limb movements in the human subthalamic nucleus
Source: Clin Neurophysiol. 2019 May;130(5):727–38. doi: 10.1016/j.clinph.2019.02.011 (PMC6487671; doi:10.1016/j.clinph.2019.02.011)
Supplement: Supplementary data 1 [file mmc1.docx]

# Supplementary Material

| **Subject** | **Sex** | **Age DBS (y)** | **Disease Duration (y)** | **Dominant Symptom** | **MDS-UPDRS OFF** | **MDS-UPDRS ON** | **Num Hem** | **STN site** | **Limb assessment** |
| --- | --- | --- | --- | --- | --- | --- | --- | --- | --- |
| 1 | m | 49 | 8 | akinetic rigid, left | 36 | 14 | 1 | L | ContraLat, IpsiLat |
|  |  |  |  |  |  |  | 2 | R | ContraLat, IpsiLat |
| 2 | m | 55 | 3 | akinetic-rigid right | 40 | 25 | 3 | L | ContraLat, IpsiLat |
|  |  |  |  |  |  |  | 4 | R | ContraLat, IpsiLat |
| 3 | f | 73 | 11 | akinetic-rigid right | 24 | 14 | 5 | L | ContraLat, IpsiLat |
|  |  |  |  |  |  |  | 6 | R | ContraLat, IpsiLat |
| 4 | m | 64 | 16 | akinetic-rigid, left | 56 | 23 | 7 | L | ContraLat |
|  |  |  |  |  |  |  | 8 | R | ContraLat, IpsiLat |
| 5 | m | 53 | 6 | akinetic-rigid, left | 30 | 13 | 9 | L | ContraLat |
| 6 | m | 34 | 7 | akinetic-rigid, right | 29 | 10 | 10 | L | ContraLat, IpsiLat |
| 7 | m | 54 | 11 | akinetic-rigid, left | 61 | 27 | 11 | L | ContraLat, IpsiLat |
|  |  |  |  |  |  |  | 12 | R | ContraLat, IpsiLat |
| 8 | m | 61 | 9 | akinetic-rigid, right | 30 | 6 | 13 | L | ContraLat |
|  |  |  |  |  |  |  | 14 | R | ContraLat |
| 9 | m | 72 | 9 | tremor dominant, right | 50 | 14 | 15 | L | ContraLat |
|  |  |  |  |  |  |  | 16 | R | ContraLat |
| 10 | f | 53 | 5 | akinetic rigid, right | 27 | 21 | 17 | R | ContraLat, IpsiLat |
| 11 | m | 64 | 13 | akinetic rigid, left | 40 | 7 | 18 | L | ContraLat, IpsiLat |
|  |  |  |  |  |  |  | 19 | R | ContraLat |
| 12 | f | 70 | 9 | tremor dominant, right | 30 | 6 | 20 | R | ContraLat, IpsiLat |
| **Mean ± SEM** | m(9), f(3) | 58.5 ± 3.2 | 8.9 ± 1.0 |  | 37.8 ± 3.5 | 15.0 ±2.1 |  |  |  |

**Supplementary Table 1. Clinical details.** Num = number; Hem = hemisphere; R = right; L = left; y = years; m = male; f= female; MDS-UPDRS = Movement Disorder Society - Unified Parkinson’s disease rating scale Part III; SEM = standard error of the mean.

**Supplementary Figure 1. Resting Spectrum.** Shows the averaged amplitude-frequency spectrum at rest and across hemispheres. The amplitude in the beta frequencies (13-35Hz) is increased, as it is typically case in patients with Parkinson’s disease off their dopaminergic medication. In addition, the figure indicates a double bump of the amplitude in the lower (13-20Hz) and upper (21-35Hz) beta frequency range. Values are illustrated as Mean ± SEM.


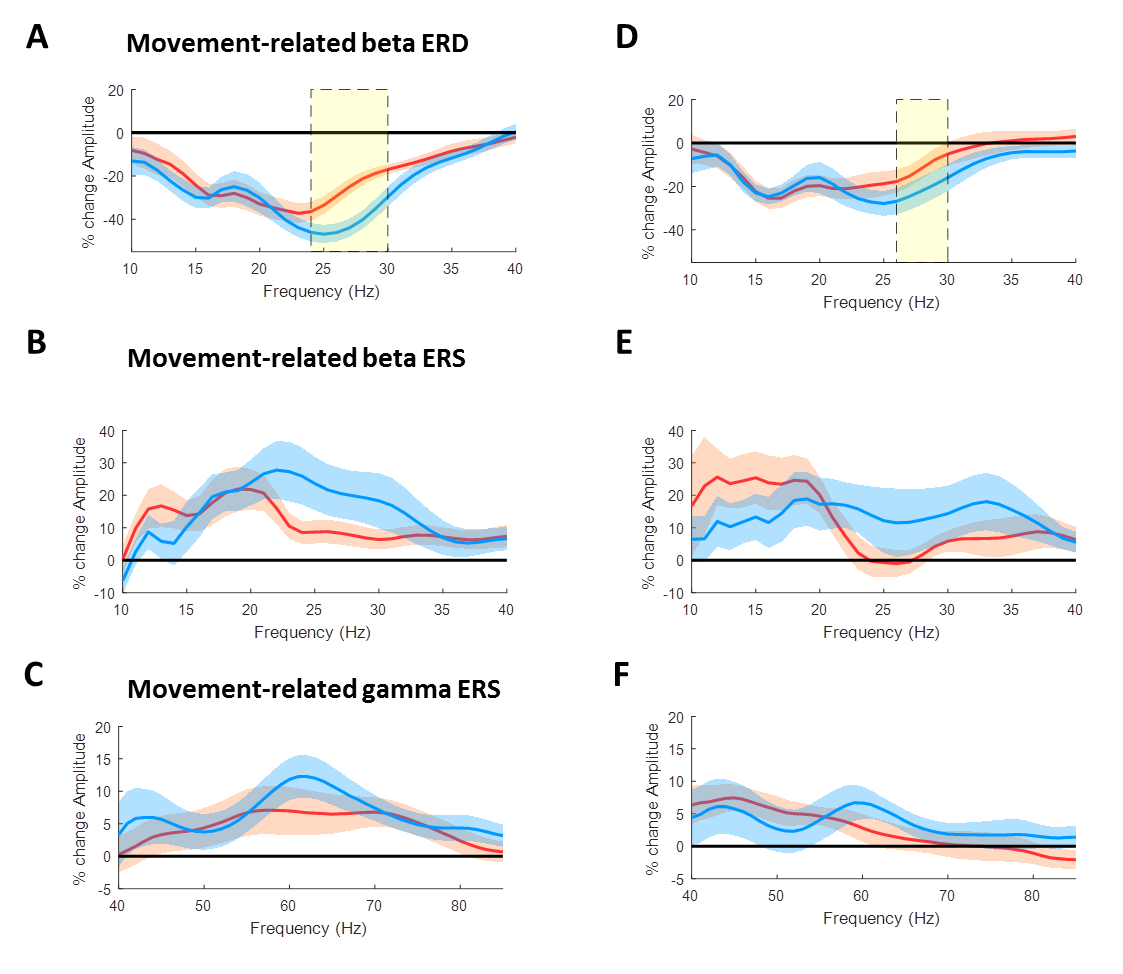


**Supplementary Figure 2. Beta ERD/ERS and gamma ERS related to contralateral and ipsilateral upper and lower limb movements with group adjusted time windows.** Here, to determine the movement related spectral changes for both contralateral (A,B,C) and ipsilateral movements (D,E,F), a 300ms time window was applied around the maximum averaged beta ERD (A,D), beta ERS (B,E) and gamma ERS (C,F), separately for upper and lower limbs. Similar as in previous results (figures 2, 4, 5, 6), where a common time-window for upper and lower limb movements was set (see method section), the key results are similar. Both beta ERD for contra and ipsilateral movements show an increased involvement at higher beta frequencies in the cluster-based permutation test (A,D), the beta ERS still shows a trend of increased involvement at higher beta frequencies (B,E), while no significant difference was found for the gamma ERS. In overall, this additional illustration supports the robustness of findings, as they are true for various methodological approaches. Values are illustrated as Mean ± SEM.
